# Exemplar Hospital initiation trial to Enhance Treatment Engagement (EXHIT ENTRE): protocol for CTN-0098B a randomized implementation study to support hospitals in caring for patients with opioid use disorder

**DOI:** 10.1186/s13722-024-00455-9

**Published:** 2024-04-11

**Authors:** Gavin Bart, P. Todd Korthuis, Julie M. Donohue, Hildi J. Hagedorn, Dave H. Gustafson, Angela R. Bazzi, Eva Enns, Jennifer McNeely, Udi E. Ghitza, Kara M. Magane, Paulette Baukol, Ashley Vena, Jacklyn Harris, Delia Voronca, Richard Saitz

**Affiliations:** 1 Department of Medicine, Hennepin Healthcare and University of Minnesota, 701 Park Avenue, Minneapolis, MN 55415 USA; 2 Department of Medicine, Addiction Medicine Section, Oregon Health & Science University, 3181 SW Sam Jackson Park Rd, 97239-3098 Portland, OR USA; 3 Department of Health Policy and Management, University of Pittsburgh School of Public Health, Pittsburgh, PA 15261 USA; 4 Center for Care Delivery & Outcomes Research, Minneapolis VA Health Care System, University of Minnesota, 1 Veterans Drive, Minneapolis, MN 55417 USA; 5 Center for Health Enhancement Systems Studies, University of Wisconsin, 1513 University Ave., Madison, WI 53706 USA; 6 Herbert Wertheim School of Public Health, University of California, San Diego; La Jolla, CA USA; 7 Boston University School of Public Health, 801 Massachusetts Ave, Suite 431, Boston, MA 02118 USA; 8 Division of Health Policy and Management, University of Minnesota School of Public Health, 420 Delaware St. SE, Minneapolis, MN 55408 USA; 9 Department of Population Health, Section on Alcohol, Tobacco and Drug Use, NYU School of Medicine, 180 Madison Avenue, 17th floor, New York, NY 10016 USA; 10 Department of Medicine, Division of General Internal Medicine and Clinical Innovation, NYU School of Medicine, 462 1st Avenue, New York, NY 10016 USA; 11 National Institute on Drug Abuse (NIDA) Center for the Clinical Trials Network (CCTN), Bethesda, MD 20892 USA; 12 Berman Center for Outcomes & Clinical Research, 701 Park Ave, Ste. PP7.700, Minneapolis, MN 55415 USA; 13 The Emmes Company, LLC, 401 N. Washington St. #700, Rockville, MD 20850 USA; 14 Currently: Regeneron Pharmaceuticals, Inc, 777 Old Saw Mill River Rd, Tarrytown, Deceased, NY 10591-6707 USA

**Keywords:** Opioid use disorder, Medications for opioid use disorder, Implementation, Protocol, Practice facilitation

## Abstract

**Background:**

Hospitalizations involving opioid use disorder (OUD) are increasing. Medications for opioid use disorder (MOUD) reduce mortality and acute care utilization. Hospitalization is a reachable moment for initiating MOUD and arranging for ongoing MOUD engagement following hospital discharge. Despite existing quality metrics for MOUD initiation and engagement, few hospitals provide hospital based opioid treatment (HBOT). This protocol describes a cluster-randomized hybrid type-2 implementation study comparing low-intensity and high-intensity implementation support strategies to help community hospitals implement HBOT.

**Methods:**

Four state implementation hubs with expertise in initiating HBOT programs will provide implementation support to 24 community hospitals (6 hospitals/hub) interested in starting HBOT. Community hospitals will be randomized to 24-months of either a low-intensity intervention (distribution of an HBOT best-practice manual, a lecture series based on the manual, referral to publicly available resources, and on-demand technical assistance) or a high-intensity intervention (the low-intensity intervention plus funding for a hospital HBOT champion and regular practice facilitation sessions with an expert hub). The primary efficacy outcome, adapted from the National Committee on Quality Assurance, is the proportion of patients engaged in MOUD 34-days following hospital discharge. Secondary and exploratory outcomes include acute care utilization, non-fatal overdose, death, MOUD engagement at various time points, hospital length of stay, and discharges against medical advice. Primary, secondary, and exploratory outcomes will be derived from state Medicaid data. Implementation outcomes, barriers, and facilitators are assessed via longitudinal surveys, qualitative interviews, practice facilitation contact logs, and HBOT sustainability metrics. We hypothesize that the proportion of patients receiving care at hospitals randomized to the high-intensity arm will have greater MOUD engagement following hospital discharge.

**Discussion:**

Initiation of MOUD during hospitalization improves MOUD engagement post hospitalization. Few studies, however, have tested different implementation strategies on HBOT uptake, outcome, and sustainability and only one to date has tested implementation of a specific type of HBOT (addiction consultation services). This cluster-randomized study comparing different intensities of HBOT implementation support will inform hospitals and policymakers in identifying effective strategies for promoting HBOT dissemination and adoption in community hospitals.

**Trial registration:**

NCT04921787.

**Supplementary Information:**

The online version contains supplementary material available at 10.1186/s13722-024-00455-9.

## Introduction

Hospitalizations attributable to opioids have increased more than 50% over the past decade with an estimated annual cost of $700 million [1–4]. In addition to opioid overdose-related hospitalizations, opioid-related infectious endocarditis, skin and soft tissue infections, and viral hepatitis hospitalizations are significantly increasing [5–7]. The rate of inpatient opioid-related hospitalizations (224.6 per 100,000 stays) exceeds the rate of opioid-related emergency department (ED) encounters (177.7 per 100,000 visits) [1]. Patients with opioid-related hospitalizations rarely receive effective opioid use disorder (OUD) treatment, which is grounded in the use of medications for opioid use disorder (MOUD) such as methadone and buprenorphine [8, 9]. As a result of un- or under-treated OUD, withdrawal, and craving, patients often leave before medically advised (i.e., against medical advice), not completing treatment for the acute condition for which they were hospitalized, return to opioid use, and are at increased risk for opioid overdose death [10–12]. Although MOUDs are efficacious, how to best implement MOUD in the hospital setting and continue them following discharge remains unclear.

Hospitalization represents a reachable moment for patients with OUD [13–16]. While most medical and surgical inpatients with OUD may not come to the hospital seeking addiction treatment, patient surveys indicate that two-thirds want to stop using opioids and half want to start taking MOUD [14, 17–21]. Initiating hospital-based opioid treatment (HBOT) in the hospital setting is feasible [22, 23] and associated with better outcomes, including decreased emergency services utilization [24], increased completion of medical therapy [25], reduced substance use [16, 26] and improved linkage to outpatient addiction treatment [16, 24, 27]. While some hospitals have formed addiction consultation services to take on this role, this approach is resource intensive, may not be feasible in some settings, and may not be necessary for effective HBOT [28]. 

There are significant barriers to HBOT and MOUD treatment linkage following hospital discharge. Barriers to inpatient MOUD initiation include the lack of clinicians with addiction expertise, clinicians’ unwillingness to obtain expertise, limited availability of outpatient providers and programs to accept these patients, lack of insurance coverage, and federal privacy regulations that can limit coordinating and integrating medical and addiction care [29]. Clinicians who are willing to prescribe buprenorphine at the time of hospital discharge often prescribe only a short (e.g., 5–7 days) supply of MOUD to bridge the gap between hospital discharge and entry into community-based treatment [16]. Yet, in a randomized trial linking hospitalized patients with OUD into community-based treatment, the median time to entrance into OUD treatment was 16 days, even with facilitated linkage [16]. This gap in medication supply may, in part, explain why only 39% of patients started on sublingual buprenorphine in the hospital were engaged in treatment at 30-days post discharge [27]. Systematic efficacy reviews, national quality measures, and practice guidelines for MOUD initiation and engagement exist but, have been inadequate for improving care [30–34]. 

Implementation science can identify optimal strategies for translating evidence-based treatments into practice. Promoting adoption of HBOT requires addressing organizational and individual clinician practice [35]. Implementation strategies range from simple and discrete (e.g., educational meetings, a clinician manual with checklists), to combinations (e.g., training and technical assistance (TTA)), to blended strategies (such as the high-intensity strategy utilized in this study) that address multiple barriers [36]. Selecting appropriate strategies involves choosing those that address barriers, recognize specific challenges (e.g., adoption and scale up, in this case), and include identifying target actors (e.g., hospitals and their administrative and clinical staff), actions (e.g., consultations), and implementation outcomes (e.g., MOUD initiation and engagement for all with OUD).

This study tests two implementation strategies for promoting adoption of HBOT in community hospitals that have expressed interest in addressing OUD. Specifically, this cluster-randomized hybrid type-2 implementation trial [37] will test two levels of implementation: (1) training and education only (i.e., low-intensity) versus (2) training and education plus practice facilitation (i.e., high-intensity). This study stands in contrast to that of McNeely et al. [38], which tested the implementation of a specific model of HBOT (the addiction consultation service) because we are randomizing hospitals to different levels of implementation support and are not testing a specific model of HBOT, as hospitals will determine their preferred approach to HBOT. We hypothesize that, compared to the low-intensity strategy, the high-intensity strategy will result in a greater proportion of patients with OUD engaged in MOUD care 34-days following hospital discharge.

## Methods

### Study objectives and design

The primary objective of this study is to compare a low- versus high-intensity implementation strategy in helping community hospitals develop HBOT services that will increase engagement in MOUD care following hospital discharge. Secondary objectives include evaluating MOUD engagement during different time periods of the intervention (e.g., year 1 versus year 2) and comparing the effect of implementation strategies on additional outcomes such as ED visits, acute care hospitalizations, and discharges before medically advised. Further, we will use multiple methods (see below) to explore implementation outcomes, barriers, facilitators, and cost-effectiveness of the high-intensity versus the low-intensity intervention.

Funded by the NIH HEAL Initiative® and conducted through a cooperative agreement in the National Institute on Drug Abuse National Drug Abuse Treatment Clinical Trials Network (CTN), CTN-0098B Exemplar Hospital Initiation Trial to Enhance Treatment Engagement (EXHIT ENTRE) is a cluster-randomized hybrid type-2 implementation study where the unit of randomization is the community hospital. Four sites in four states (MA, MN, NY, and OR) (hereafter referred to as hubs) with expertise in HBOT implementation will facilitate implementation in 24 community hospitals (6 community hospitals per hub) randomized in a blocked 1:1 ratio to either 24-months of low- or high-intensity implementation support. The low-intensity arm will receive a best-practices manual, one 60-minute orientation to the manual, a video webinar series on HBOT topics, and reactive response by the hub team to community site questions. Hospitals randomized to the high-intensity arm will receive low-intensity interventions plus approximately monthly practice facilitation support from the hub as well as 10% effort funding for a local champion, regularly scheduled videoconferences with the hub, and tele-mentoring over 24-months.

**Fig. 1:**
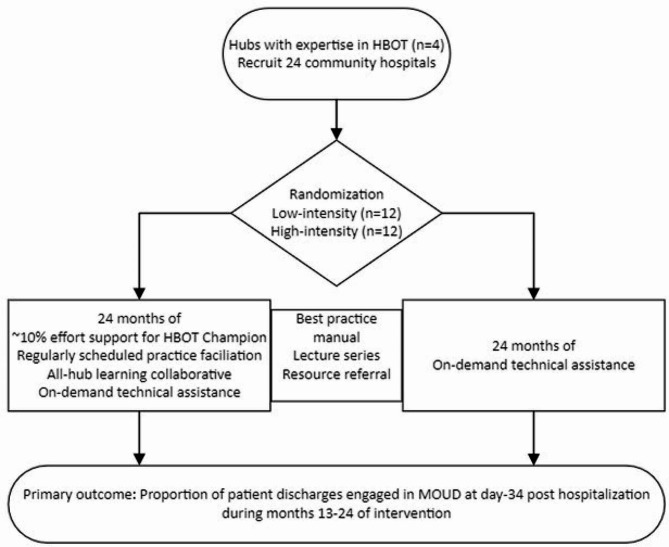
Study schema

This facilitation uses a program planning model approach to empower hospitals to lead and shape their approach to HBOT while utilizing the hub experts as advisors and mentors [39]. Structured facilitation includes a 20-minute didactic presentation by the hub team focused on a topic relevant to a hospital’s implementation stage, followed by a presentation by one of the site champions, who will present and discuss an implementation challenge or success. The hub team will provide immediate guidance and technical assistance on addressing challenges and use the information to prepare didactic content for the next session. Sessions will include detailed review of the HBOT manual topics tailored to the hospital-specific barriers and facilitators that champions are facing. The overall content of practice facilitation sessions will be flexible, guided by the needs of the hospital and staff. This flexibility allows the intervention to focus on supporting hospitals in local areas of concern rather than those predefined by the intervention team. The sessions also serve to identify and engage key stakeholders to address OUD stigma, align HBOT with leadership priorities, and identify and measure key internal financial and quality measures. Further, national learning collaboratives will convene champions and staff across high-intensity hospitals for sharing information, case studies, and implementation approaches across hospitals and hubs.

### Hub selection

Solicitation for hospitals with expertise in implementing HBOT to provide the intervention will be distributed through the NIDA CTN. To be eligible, hub hospitals must be in states with Medicaid programs covering all FDA-approved MOUD, have access to state Medicaid claims data with no more than a 12-month lag, and be able to recruit at least six community hospitals with an aggregated average of 100 discharges per hospital per year for Medicaid patients with OUD.

### Community hospital selection

Eligible, community hospitals must:

1. Be located in the region of a hub, defined by lead investigators. Region will usually be defined as a state.
2. Provide inpatient general medical care.
3. Be willing to identify a site champion to promote and adopt change that can address OUD in hospitalized patients.
4. Have hospital personnel who state that their institution is interested in and would be willing to work to implement MOUD prior to hospital discharge.
5. Commit to having buprenorphine-waivered prescribers willing and able to write prescriptions to bridge discharged patients to post-discharge OUD treatment, or available direct entry into outpatient MOUD with methadone or buprenorphine. (Note that this protocol was developed prior to the elimination of the DATA 2000 buprenorphine waiver requirement).
6. Have hospital staff who express willingness to engage with a hub team for training and for data collection.
7. Be willing to be randomized to low-intensity or high-intensity implementation support.
8. Have sufficient numbers of Medicaid OUD discharges (any listed diagnosis; sufficient is defined as at least enough such that, when aggregated, each hub’s participating hospitals will average 100 Medicaid patient OUD discharges per year).
9. Medicaid data must capture at least 3 discharge diagnoses, outpatient MOUD, and be available within no more than 12 months of discharge.

Participating community hospitals must not:

1. Have an Addiction Consult Service (ACS) routinely prescribing MOUD at discharge.
2. Have a functioning HBOT program or be imminently starting an HBOT initiative, as confirmed by the investigator team.
3. Be a Veterans Affairs hospital.

Following randomization, champions, hospital administrators, or other personnel at the community hospitals will identify personnel determined to be relevant stakeholders for participating in HBOT implementation, surveys, and qualitative interviews. Hospital personnel determined to have in-depth knowledge of current hospital practice related to managing OUD and potential barriers and facilitators to implementing HBOT will be recruited for qualitative interviews and surveys in line with a purposive and snowball sampling strategies [40, 41]. Implementation surveys also will be solicited via QR code from audience members attending the intervention lecture series and from email announcements sent to hospital staff by site champions at the end of the intervention period.

A single independent commercial institutional review board (IRB) has approved this study and all hubs have ceded to this single IRB. In addition, this study was reviewed by an independent Data and Safety Monitoring Board (DSMB) appointed by the NIDA Center for the Clinical Trials Network (NIDA CCTN).

### Randomization

Twenty-four eligible community hospitals (clusters) will be identified and then randomized by a central data and statistical center in a 1:1 ratio stratified by hub. An even number of participating hospitals will be required for each of the 4 hubs in order to have the same number of hospitals randomized in each arm within each hub. Hubs will inform hospitals of their intervention assignment, so randomization will not be concealed after intervention assignment. When possible, investigators will remain blind to group assignment during data analysis.

### Outcomes

The primary outcome is “engagement with MOUD” measured as the proportion of community hospital OUD discharges engaged with MOUD within 34-days following hospital discharge during months 13–24 of the intervention. This outcome is adapted (to include those with a pre-existing OUD diagnosis) from the Healthcare Effectiveness Data and Information Set (HEDIS) Initiation and Engagement with Treatment (IET) measure used by more than 90% of health plans and specified by the National Committee on Quality Assurance (NCQA), making it relevant to how hospitals are rated and, potentially, reimbursed [30, 31, 38]. . Primary, secondary, and most exploratory outcomes will be derived from state Medicaid claims data (see Supplementary materials for specific claims codes that define these outcomes). Outcome measures are presented in Tables 1a and 1b.

**Table 1a:** Medicaid-based outcome measures

	Study Periods for Medicaid Data Abstraction (in months)			
	Pre-Implementation (-12 randomization (-1))	Implementation Year 1 (1–12)	Implementation Year 2 (13–24)	Post-Implementation (25–36*)
**MEASURES OF PRIMARY AND SECONDARY OUTCOMES**				
MOUD^a^ engagement 34 days post-discharge	X	X	X	X
ED^b^ visits or acute hospitalizations within 30 days post-discharge (OUD^c^ related or not)	X	X	X	X
Discharges against medical advice during index hospitalization	X	X	X	X
**MEASURES OF EXPLORATORY OUTCOMES AND OTHER ASSESSMENTS**				
Hospitalization Diagnoses (at every discharge and ED visit)	X	X	X	X
Medical Comorbidity	X	X	X	X
Non-Fatal Overdose	X	X	X	X
All-cause mortality (Medicaid data enrollment files) including time to death	X	X	X	X
Time to re-admission (ED or acute care) post- discharge	X	X	X	X
Discharges against medical advice during subsequent hospital visits	X	X	X	X
MOUD initiation	X	X	X	X
Opioid use disorder treatment initiation	X	X	X	X
Pre-hospitalization MOUD within 60, 30, 14 or 0 days before admission	X	X	X	X

a Medication for Opioid Use Disorder

b Emergency Department

c Opioid Use Disorder

**Table 1b:** Non Medicaid-based outcome measures

		Follow-up Time Points (Month)					
	0^1^	6	12	18	24	30	36
Characteristics of the community hospitals	X		X		X		X
Demographics of community hospital staff	X	X^2,3^	X^2,3^	X^2,3^	X	X^2,3^	X^2,3^
Organizational Change Manager^3^	X	X^2^	X^2^	X^2^	X	X^2^	X^2^
Sustainability Tool^3^	X	X^2^	X^2^	X^2^	X	X^2^	X^2^
Consolidated Framework for Implementation Research Inner Setting Measure	X						
Implementation outcome survey measures (AIM/IAM/FIM)	X				X		
Buprenorphine waiver assessment	X		X^4^		X^4^		X^4^
Fidelity to the Intervention^5^		X	X	X	X		
HBOT MOUD implementation: qualitative interviews	X^2^				X^6^		
Cost evaluation			X		X		
Practice Facilitators Observational Log^7^		X	X	X	X		

1 Hospital randomization day is Day 1

2 High-intensity hospitals only

3 Small group of stakeholders only

4 This was discontinued following federal removal of the waiver requirement

5 Completed quarterly during the implementation period

6 Month 24 interviews will be completed in all high-intensity hospitals and possibly low-intensity community hospitals if the month-24 OCM score is ≥ 10

7 Completed in “real-time” to document day-to-day interactions with community hospital staff during the implementation period

Additional implementation outcomes and constructs guided by the Reach, Adoption, Implementation, and Maintenance domains of the RE-AIM framework (Table 2) will be explored using quantitative and qualitative methods [42]. 

**Table 2:** RE-AIM implementation framework

RE-AIM Element	Items	Data Sources	Tools
Reach	Staff reports of offering MOUD^a^ Engagement with MOUD	Interviews Medicaid claims data	Community hospital personnel interviews.
Effectiveness	Effect of the intervention on patient outcomes	See Outcomes section of protocol.	Medicaid claims data.
Adoption	Barriers to adopting HBOT Supports that will need to be in place for hospitals to adopt the intervention Buprenorphine waivered inpatient providers	Community hospital personnel interviews and surveys.	Community hospital personnel interviews and surveys (Consolidated Framework for Implementation Research (CFIR) inner setting measure, OCM^b^, Acceptability of Intervention Measure, Intervention Appropriateness Measure, and Feasibility of Intervention Measure (AIM/IAM/FIM)). Practice Facilitators Observational Log.
Implementation	Supports needed to be in place to ensure consistent delivery of the intervention Tools needed to deliver the intervention consistently Cost of the intervention Feasibility, appropriateness and acceptability of MOUD	Survey of perceptions of staff and leadership. Survey of perceptions of site/hub experts and research staff. Community hospital personnel interviews and surveys. Cost data.	Community hospital personnel interviews and surveys (CFIR inner setting measure, OCM, AIM/IAM/FIM, Sustainability Model) Observational logs. Records of site/hub expert and staff, internal champion and staff time required. Research records regarding program costs.
Maintenance	Resources needed to maintain the intervention in the long run Adaptations needed to integrate the intervention into regular practice	Community hospital personnel interviews and surveys.	Practice Facilitator Observational Logs. Community hospital personnel interviews and surveys (OCM, Sustainability Model, AIM/IAM/FIM)

a Medication for Opioid Use Disorder

b Organizational Change Manager

### Assessments

#### Data collection and assessment

The primary, secondary, and most exploratory outcome effectiveness measures will be derived from state Medicaid claims data (Table 1). Medicaid claims were selected because Medicaid is the most common payer for opioid-related hospitalizations and for outpatient MOUD [5, 43, 44]. A distributed research network approach using a common data model will allow for standardized analyses and comparisons across states without the need for data-sharing agreements between states [45]. Medicaid data and hospital characteristic data collection are presented in supplemental materials.

For implementation measures, we will administer eight assessments to staff/personnel at each community hospital via a web-based consent and survey interface: (1) Organizational Change Manager (OCM), a validated survey of tactical considerations regarding an implementation [46], (2) Consolidated Framework for Implementation Research (CFIR) Inner Setting Measure, a valid measure of contextual factors that influence and predict implementation [47], (3) an adapted Sustainability Tool, providing a numerical prediction of sustainability potential [48, 49], 4–6) Acceptability of Intervention, Intervention Appropriateness, and Feasibility of Intervention Measures (AIM, IAM, FIM) are implementation outcome survey measures used to monitor and evaluate the success of implementation efforts [49], 7) a buprenorphine waiver assessment, and 8) a demographics survey. Survey measures address questions about the inner setting at baseline, and implementation barriers, facilitators, and outcomes at follow-up. To identify survey participants, we will identify contacts who could be involved in MOUD implementation and then, using a snowball technique [41], we will invite others including relevant leaders, supporters, detractors/skeptics, and opinion leaders. The OCM and Sustainability Tool surveys will be administered to a small group (5–10) of community hospital personnel per hospital that includes only the individuals who are closely involved in the implementation efforts, whereas the CFIR inner setting measure, and AIM/IAM/FIM surveys will be administered to a broader sample of personnel/staff/stakeholders from the organization, in addition to the smaller group (5–10) of implementation team members. Participants will be assigned unique identifiers for data tracking purposes and offered $50 compensation.

For qualitative interviews, we will use purposive and snowball sampling strategies [40, 41] to identify 3–5 individuals with diverse roles within each high-intensity hospital (e.g., physicians, physician assistants, nurse practitioners, nurses, social workers, and administrative leaders). Hospital champions will be included and asked to recommend additional individuals who will be knowledgeable about MOUD implementation readiness (including perceived local barriers and facilitators to HBOT) at month 0 and implementation processes and outcomes at month 24. Select high-performing low-intensity hospitals staff may also be interviewed at month 24 if their OCM survey results indicate a high likelihood of change. Interviewed individuals will provide verbal informed consent and be offered $50 compensation.

Qualitative interviews will be conducted by trained study personnel using a semi-structured guide developed in collaboration with qualitative research experts and each hub team, especially team members with clinical experience in HBOT. The guide will contain open-ended questions and detailed probes exploring a hospital’s approaches to treating patients with OUD, perceived barriers and facilitators to increasing MOUD prescribing (at month 0), and experiences with HBOT implementation and perspectives on the study intervention, HBOT adoption, maintenance, and sustainment. Interviews will be conducted virtually, via videoconferencing, or by phone, will last ∼ 45–60 min, and will be audio-recorded for professional transcription (with potential identifying information redacted). Prior to analysis, interviewers will review transcripts for accuracy and to confirm de-identification following a structured protocol [50]. 

Investigators will track all encounters with participating hospitals during the intervention using a practice facilitator’s observation log. The log tracks each encounter for date, type of encounter (e.g., webinar, consultation, guideline development/review), length of the encounter, number of people involved, and job type of those involved (e.g., physician, nurse, social worker). This log will be used to evaluate fidelity to the intervention and as source data for cost-estimation of the implementation intervention.

### Analyses

#### Sample size, power, and effect size

To assess power for the primary outcome comparing the high- vs. low-intensity intervention at the discharge record level, we assumed the engagement rate in low-intensity hospitals will be 20% on average, and that hospitals in an intervention strategy group vary in their engagement proportion with a standard deviation (SD) of 0.15 on the logit scale (and a corresponding intraclass correlation coefficient of 0.0067 assuming logistic regression [51]). The standard deviation of 0.15 is based on Oregon and Massachusetts Medicaid data and is conservative for our purposes because the analysis will be stratified by site/hub and will adjust for baseline engagement rate, which will reduce variation between hospitals within strata, and this standard deviation does not account for those reductions. With 12 community hospitals per intervention arm and 100 patient discharges per community hospital, we have 85% power (two-sided test, alpha = 0.05) to detect a treatment effect with odds ratio of 1.45. Using the same assumptions as above, for a comparison of high- vs. low-intensity arms at the hospital level (i.e., no cluster effect which means interclass correlation coefficient is not different from zero) we have 97% power to detect a treatment effect with odds ratio of 1.45.

Baseline (prior to randomization) descriptive characteristics will be summarized by group, stratified by hub/state, at the community hospital level as well as at the participant discharge record level. Descriptive summaries of the distribution of continuous baseline variables will be presented with percentiles (median, 25th and 75th percentiles), and with mean and standard deviation. Categorical variables will be summarized in terms of frequencies and percentages.

#### Effectiveness analyses

The analysis of the primary outcome will be intention to treat based on randomized assignment and will compare high- and low-intensity arms using a mixed-effects logistic regression analysis. Hospitals that drop out of study participation will not be replaced and data will be tracked per randomized assignment. The main analysis model for the primary outcome will include hub as a hierarchical nested random effect with hospitals nested in the hubs. Observations are individual patient hospital discharges aggregated at hospital level, the primary outcome (derived from state Medicaid claims data) is patient MOUD engagement 34-days after hospital discharge (yes/no), the random effects are hubs and community hospitals, with hospitals nested within sites/hubs, and the fixed effect is strategy for MOUD engagement.

Analyses of secondary outcomes will have the same basic form as the primary analysis, with differences arising from the type of outcome (binary vs. interval-scaled vs. count) and from inclusion of further adjusters. Survival analysis techniques may be used for outcomes such as time to readmission or death [52]. 

#### Implementation analyses

Scores for AIM/IAM/FIM, OCM, CFIR inner setting measure, and Sustainability Tool will be calculated in accordance with published guidance and compared both across hospital groups and over time. The Sustainability Tool includes weighted factors with scores that are combined in an additive multi-attribute utility model [53] Analysis of the OCM uses a Bayesian model where a likelihood ratio is assigned to each possible response to each question. The likelihood ratios for the selected responses and the prior odds are multiplied together to yield *a posterior* odds, which can be averaged across the individual respondents. Group by time interaction analyses will be used to assess the association between changes in these measures through time and the primary and secondary quantitative outcomes.

The RE-AIM framework will be used to identify the reach of our implementation intervention, its efficacy, adoption, implementation, and maintenance, or sustainability [54]. This framework is well suited for randomized interventions in real-world settings.

#### Qualitative analyses

We will use a collaborative codebook development process involving a lead qualitative investigator and interviewers from each hub following an iterative, collaborative codebook development process [55, 56]. Parent- and sub- codes will relate to defined theoretical constructs (e.g., organizational readiness to change, resources, local environment, staffing models) and emergent topics identified in the data. After reaching consensus on final codes and definitions, we will apply codes to transcribed interview data using NVivo qualitative research management software (Lumivero, Denver, CO) in preparation for thematic analysis [57]. 

#### Economic analyses

The purpose of the economic evaluation will be to assess the value, in terms of both costs and effectiveness, of implementation support for HBOT in community hospitals. Here, costs, collected as resource use (e.g., personnel) rather than dollar amount spent, will consist of the costs of the different components of the provided implementation support. These costs will be divided into one-time setup costs and ongoing maintenance costs. Only maintenance costs will be used in calculating the efficiency of the implementation programs, as these are the costs that would be expended if the implementation programs were to be scaled up beyond the trial population. Resource use categories will consist of the time spent by hub staff providing training and support to community hospitals, as well as the time use of each local community hospital champion (for sites receiving the high-intensity intervention). Hub staff will document time spent on the preparation and delivery of each training session and will also document each supportive interaction with community hospitals, including approximate duration. The cost of personnel time will be estimated as the average total compensation for the appropriate job class. Effectiveness will be measured in terms of the change in proportion of patients engaged in MOUD at discharge and changes in post-discharge healthcare utilization (both acute care and hospital readmissions). The overall value of a given intensity of implementation support will then be measured in terms of the additional cost per additional unit increase in patients engaged in MOUD at discharge, and secondarily as the cost per acute healthcare visit averted and/or hospital readmission averted.

### Data safety and monitoring

An independent CTN Data Safety and Monitoring Board (DSMB) will examine accumulating data at least annually to assure the study’s scientific goals are being met. It will determine whether there is support for continuation of the trial, or evidence that study procedures should be changed, or if the trial should be halted, or if there is inadequate trial performance. There will not be any safety monitoring or interim analyses for this study, thus stopping parameters will be based on priorities of the funder.

### Trial status

Following the development of the study protocol, the federal requirement for obtaining a waiver to prescribe buprenorphine for OUD has been removed. As such, the protocol was amended to cease collection of data about the number of buprenorphine waivered providers at each hospital.

## Discussion

We describe the protocol for a cluster-randomized hybrid type-2 implementation science trial comparing two approaches to support hospitals in developing hospital based opioid treatment programs. While a few retrospective analyses and clinical trials have shown HBOT to be effective in engaging patients in post-hospitalization MOUD and in reducing acute care utilization, studies of HBOT implementation are lacking. McNeely et al. completed a stepped-wedge hybrid type-1 implementation study of a specific model of HBOT (the addiction consultation service) [38], but there are no randomized studies evaluating the impact of distinct levels of HBOT implementation support on patient outcomes. By using a hybrid type-2 design measuring both implementation and effectiveness [37], we will determine the intensity required to successfully establish and support HBOT services at community hospitals. Our mixed-methods approach is designed to help understand the implementation processes and contexts, and to help explain the primary and secondary outcomes. Study findings may inform policymakers and funders in choosing effective strategies for promoting HBOT adoption in community hospitals.

The primary outcome measure of MOUD engagement following hospital discharge is based on a national quality metric [30], thus having general acceptance by hospitals as being relevant and important. Our use of Medicaid claims data for the primary and most secondary outcome measures mirrors how several quality metrics are tracked and, for some, incentivized. A disproportionate number of hospitalized patients with OUD are covered by Medicaid and our ability to compare these patients with all other Medicaid patients seen within participating hospitals allows us to accrue larger sample sizes, over a shorter period of time, and at lower expense than could be achieved through individual patient enrollment and outcome tracking. Further, the large sample size likely will allow for a variety of sensitivity analyses to help identify optimal characteristics associated with MOUD engagement.

While the hybrid type-2 design allows us to assess both patient level outcomes and compare implementation approaches in effecting these outcomes, there are limitations to our approach. While our intervention is designed such that hospitals can choose their preferred approach to HBOT, it likely does not provide an adequate level of support to facilitate the implementation of a well-functioning addiction consultation service. Patient level outcomes are based on state Medicaid claims data, which may not generalize to hospitalized patients with OUD covered through other mechanisms or in states without Medicaid expansion. Our mixed methods approach should also help us explain any difference (or lack thereof) in primary and secondary outcome measures between the intervention arms. Further, OUD diagnostic codes may not adequately capture all patients with OUD, those receiving MOUD while hospitalized, or those receiving MOUD following hospital discharge [58]. Importantly, claims do not capture patient preference or choice (e.g., some may be offered but decline MOUD). There are also several different approaches to implementation and our selection of practice facilitation using a program planning model cannot be compared to a conventional (i.e., top-down) planning model or other approaches [59]. We use four expert hubs working in parallel with a common set of HBOT materials for implementation support and, therefore, are not evaluating the use of a single centralized implementation support center. Most of our implementation outcomes are tracked using the RE-AIM framework and may not adequately capture external barriers and facilitators to implementation, although qualitative interviews will explore inner and outer contextual factors that could influence implementation processes or outcomes.

In summary, the development of national quality metrics related to engagement of hospitalized patients with substance use disorders, would indicate that there is consensus that HBOT is important. Yet, performance metrics alone have been insufficient in improving the management of OUD. For example, in 2004, initiation of and engagement in addiction treatment was 12% in Medicaid nationally; in 2016 it was still 12% [31]. Establishing effective implementation strategies for HBOT at community hospitals can drive translation and dissemination of HBOT nationwide while serving as a potential model for similarly addressing other substance use disorders.

### Electronic supplementary material

Below is the link to the electronic supplementary material.

Supplementary Material 1

Supplementary Material 2

## Data Availability

No datasets were generated or analysed during the current study.

## References

[1] Weiss AJ (2016). Opioid-related inpatient stays and Emergency Department visits by State, 2009–2014.

[2] Hsu DJ (2017). Hospitalizations, costs and outcomes associated with heroin and prescription opioid overdoses in the United States 2001-12. Addiction.

[3] Unick GJ, Ciccarone D (2017). US regional and demographic differences in prescription opioid and heroin-related overdose hospitalizations. Int J Drug Policy.

[4] Winkelman TA (2018). Evaluation of amphetamine-related hospitalizations and associated clinical outcomes and costs in the United States. JAMA Netw Open.

[5] Ronan MV, Herzig SJ (2016). Hospitalizations related to opioid Abuse/Dependence and Associated Serious infections increased sharply, 2002-12. Health Aff (Millwood).

[6] Wurcel AG (2016). Increasing infectious endocarditis admissions among Young people who inject drugs. Open Forum Infect Dis.

[7] Njoroge LW (2018). Changes in the Association of rising infective endocarditis with mortality in people who inject drugs. JAMA Cardiol.

[8] Jicha C (2019). Substance Use Disorder Assessment, diagnosis, and management for patients hospitalized with severe infections due to Injection Drug Use. J Addict Med.

[9] Priest KC (2020). Opioid agonist therapy during hospitalization within the Veterans Health Administration: a pragmatic retrospective cohort analysis. J Gen Intern Med.

[10] Zhu H, Wu L-T (2019). Discharge against medical advice from hospitalizations for substance use disorders: the potential impact of the Affordable Care Act. Drug Alcohol Depend.

[11] Thakrar AP (2023). Trends in before medically advised discharges for patients with opioid Use Disorder, 2016–2020. JAMA.

[12] Larochelle MR (2019). Touchpoints - opportunities to predict and prevent opioid overdose: a cohort study. Drug Alcohol Depend.

[13] Ducharme LJ, Chandler RK, Harris AH (2016). Implementing effective substance abuse treatments in General Medical settings: mapping the Research Terrain. J Subst Abuse Treat.

[14] Englander H (2017). Planning and Designing the Improving Addiction Care Team (IMPACT) for hospitalized adults with Substance Use Disorder. J Hosp Med.

[15] Velez CM (2017). It’s been an experience, a life learning experience: a qualitative study of hospitalized patients with Substance Use disorders. J Gen Intern Med.

[16] Liebschutz JM, Crooks D, Herman D (2014). Buprenorphine treatment for hospitalized, opioid-dependent patients: a randomized clinical trial. JAMA Intern Med.

[17] Pollini RA (2006). Does this patient really want treatment? Factors associated with baseline and evolving readiness for change among hospitalized substance using adults interested in treatment. Addict Behav.

[18] McNeely J (2012). Estimating the prevalence of illicit opioid use in New York City using multiple data sources. BMC Public Health.

[19] Rosenthal ES (2016). Suboptimal addiction interventions for patients hospitalized with Injection Drug Use-Associated Infective Endocarditis. Am J Med.

[20] Smothers BA, Yahr HT, Ruhl CE (2004). Detection of alcohol use disorders in general hospital admissions in the United States. Arch Intern Med.

[21] Naeger S (2016). Post-discharge Treatment Engagement among patients with an opioid-use disorder. J Subst Abuse Treat.

[22] Shanahan CW (2010). A transitional opioid program to engage hospitalized drug users. J Gen Intern Med.

[23] Christian N (2021). Hospital Buprenorphine Program for Opioid Use Disorder is Associated with increased inpatient and outpatient addiction treatment. J Hosp Med.

[24] Gryczynski J (2021). Preventing hospital readmission for patients with Comorbid Substance Use Disorder: a Randomized Trial. Ann Intern Med.

[25] Fanucchi LC (2020). Outpatient parenteral antimicrobial therapy plus buprenorphine for opioid use disorder and severe injection-related infections. Clin Infect Dis.

[26] Wakeman SE (2017). Inpatient Addiction Consultation for hospitalized patients increases Post-discharge abstinence and reduces addiction severity. J Gen Intern Med.

[27] Trowbridge P (2017). Addiction consultation services - linking hospitalized patients to outpatient addiction treatment. J Subst Abuse Treat.

[28] Englander H (2022). A taxonomy of Hospital-based Addiction Care models: a scoping review and key informant interviews. J Gen Intern Med.

[29] Fanucchi L, Lofwall MR (2016). Putting parity into practice - integrating opioid-use disorder treatment into the hospital setting. N Engl J Med.

[30] National Committee on Quality Assurance. Initiation and engagement of alcohol and other drug dependence treatment: The HEDIS Measure. 2017; Available from: https://www.ncqa.org/hedis/measures/initiation-and-engagement-of-alcohol-and-other-drug-abuse-or-dependence-treatment/.

[31] National Committee on Quality Assurance. NCQA updates quality measures for HEDIS2018 technical specifications update. 2017. 2019; Available from: Available from: https://www.ncqa.org/news/ncqa-updates-quality-measures-for-hedis-2018-technical-specifications-update/.

[32] Kampman K, Jarvis M (2015). American Society of Addiction Medicine (ASAM) National Practice Guideline for the Use of medications in the treatment of Addiction Involving Opioid Use. J Addict Med.

[33] Mattick RP (2009). *Methadone maintenance therapy versus no opioid replacement therapy for opioid dependence* Mattick.Richard. Davoli.Marina.Methadone.maintenance.therapy versus.no opioid.replacement.therapy for opioid.dependence.Cochrane.Database.of Systematic.Reviews: reviews 2009.Issue.3 John.

[34] Mattick RP et al. Buprenorphine maintenance versus placebo or methadone maintenance for opioid dependence. Cochrane Database Syst Rev, 2014(2): p. CD002207. 10.1002/14651858.CD002207.pub4 PMC10617756 24500948

[35] Proctor EK (2009). Implementation research in mental health services: an emerging science with conceptual, methodological, and training challenges. Adm Policy Ment Health.

[36] Powell BJ (2012). A compilation of strategies for implementing clinical innovations in health and mental health. Med Care Res Rev.

[37] Curran GM (2012). Effectiveness-implementation hybrid designs: combining elements of clinical effectiveness and implementation research to enhance public health impact. Med Care.

[38] McNeely J, et al. Study protocol for a pragmatic trial of the Consult for Addiction Treatment and Care in hospitals (CATCH) model for engaging patients in opioid use disorder treatment. Volume 14. Addiction Science & Clinical Practice; 2019. p. 5. 1. 10.1186/s13722-019-0135-7 PMC6380041 30777122

[39] Research to practice in addiction treatment: key terms and a field-driven model of technology transfer. J Subst Abuse Treat, 2011. 41(2): p. 169–78. 10.1016/j.jsat.2011.02.006 21466943

[40] Johnson JC. Selecting Ethnographic informants. SAGE; 1990.

[41] Biernacki P, Waldorf D. Snowball Sampling: problems and techniques of Chain Referral Sampling. Volume 10. Sociological Methods & Research; 1981. pp. 141–63. 2.

[42] Proctor E (2011). Outcomes for implementation research: conceptual distinctions, Measurement challenges, and Research Agenda. Adm Policy Mental Health Mental Health Serv Res.

[43] Bedi P (2021). Pattern and burden of opioid-related hospitalizations in the USA from 2016 to 2018. Br J Clin Pharmacol.

[44] The Medicaid Outcomes Distributed Research Network (2021). Use of medications for Treatment of Opioid Use Disorder among US Medicaid Enrollees in 11 States, 2014–2018. JAMA.

[45] Zivin K (2022). Design, implementation, and evolution of the Medicaid Outcomes Distributed Research Network (MODRN). Med Care.

[46] Gustafson DH (2003). Developing and testing a model to predict outcomes of organizational change. Health Serv Res.

[47] Fernandez ME (2018). Developing measures to assess constructs from the Inner setting domain of the Consolidated Framework for Implementation Research. Implement Sci.

[48] Maher L, Gustafson D, Evans A. *NHS Sustainability Model*. 2010; Available from: https://webarchive.nationalarchives.gov.uk/20160805122935/http://www.nhsiq.nhs.uk/media/2757778/nhs_sustainability_model_-_february_2010_1_.pdf.

[49] Weiner BJ (2017). Psychometric assessment of three newly developed implementation outcome measures. Implement Sci.

[50] McLellan E, MacQueen KM, Neidig JL (2003). Beyond the qualitative interview: Data Preparation and transcription. Field Methods.

[51] Goldstein H, Browne W, Rasbash J (2002). Partitioning variation in multilevel models. Underst Stat.

[52] Yoshida K (2018). Comparison of privacy-protecting analytic and data-sharing methods: a simulation study. Pharmacoepidemiol Drug Saf.

[53] Gustafson DH, Cats-Baril WL, Alemi F. Systems to support Health Policy Analysis: theory, models, and uses. Health Administration; 1992.

[54] Glasgow RE, Vogt TM, Boles SM (1999). Evaluating the public health impact of health promotion interventions: the RE-AIM framework. Am J Public Health.

[55] DeCuir-Gunby JT, Marshall PL, McCulloch AW (2011). Developing and using a codebook for the analysis of interview data: an Example from a Professional Development Research Project. Field Methods.

[56] MacQueen KM (1998). Codebook Development for Team-based qualitative analysis. CAM J.

[57] Ryan GW, Bernard HR (2003). Techniques to identify themes. Field Methods.

[58] Howell BA (2021). Validity of Incident Opioid Use Disorder (OUD) diagnoses in Administrative Data: a Chart Verification Study. J Gen Intern Med.

[59] Van de Ven AH (1980). Problem solving, planning, and Innovation. Part I. Test of the Program Planning Model. Hum Relat.

