# Supplementary material for: Exemplar Hospital initiation trial to Enhance Treatment Engagement (EXHIT ENTRE): protocol for CTN-0098B a randomized implementation study to support hospitals in caring for patients with opioid use disorder
Source: Addict Sci Clin Pract. 2024 Apr 11;19:29. doi: 10.1186/s13722-024-00455-9 (PMC11007900; doi:10.1186/s13722-024-00455-9)
Supplement: Supplementary file 2 — Supplementary Material 2 [file 13722_2024_455_MOESM2_ESM.docx]

Supplemental materials for Exemplar Hospital Initiation Trial to Enhance Treatment Engagement

(EXHIT ENTRE): protocol for CTN-0098B a randomized implementation study to support hospitals in caring for patients with opioid use disorder

1. Primary outcome analysis plan
2. Secondary and exploratory Medicaid analyses plan
3. Demographics survey

**Analysis Plan: MOUD Engagement within 34 Days Post-Discharge**

**November 18, 2022**

Description: the proportion of community hospital OUD discharges engaged with MOUD within 34 days following hospital discharge during months 13-24 of the intervention

The measure score is also calculated for each month of pre-implementation (-12 randomization (-1)), implementation Year 1 (1-12), implementation Year 2 (13-24), and post implementation (25-36*).

**DENOMINATOR:** Hospital discharges with OUD diagnosis from all non-dual, full-benefit Medicaid enrollees who are 18-64 years of age

CREATE DENOMINATOR:

1. For months 13-24 of the intervention, identify non-dual, full-benefit Medicaid enrollees who are 18-64 years of age for the duration of the year

*Note: This is consistent with the Common data Model (CDM), which only includes non-dual, full-benefit Medicaid enrollees who are 18 years of age and older for the duration of the year.*

1. For individuals identified in Step 1, identify all inpatient encounters with any diagnosis (all diagnosis fields) of OUD at any time during the measurement period.

The ICD-10 diagnosis codes used to identify OUD included:

• F11.1x, F11.2x, F11.9x, T40.0x-T40.4x and T40.6x (including selected derivative codes of those (x))

1. Of inpatient encounters identified in Step 2, exclude those billed from a non-participating hospital.
2. Identify unique hospital stays. We’ll treat inpatient claims as a single hospital stay if they are from same hospital AND the DOS_END from the first stay precedes the DOS_START to a subsequent stay by one calendar day or less.
3. Exclude hospital stays where the enrollee died during the stay.
4. Exclude hospital stays if the enrollee had any remaining days of supply for MOUD in the 14 days prior to hospital stay.
5. Check continuous enrollment from date of discharge to 33 days after hospital discharge (total of 34 days). Exclude those without the continuous enrollment.
6. Flag if there is a direct transfer to non-participating hospital after hospital stay at a participating hospital. A direct transfer is when the discharge date from the initial stay precedes the admission date to a subsequent stay by one calendar day or less.
7. Count the number of hospital discharges with OUD diagnosis per unit of period. This is the denominator.
8. As a sensitivity analysis, include all hospital stays in participating hospitals for enrollees with a diagnosis of OUD on any type of claim within 6 months before the hospital stay.

**NUMERATOR:** Hospital OUD discharges engaged with MOUD within 34 days following hospital discharge

CREATE NUMERATOR:

1. Of observations in the denominator, identify those with at least one MOUD claim within 34 days, beginning on the day of hospital discharge: (These NDC/HCPCS codes are contained in the excel file called *“MODRN_MOUD_plus_NQF3175_2021ver.xlsx”.*)
   - - Buprenorphine
     - Injectable Naltrexone
     - Buprenorphine and Naloxone

Or a HCPCS code for any of the following OUD medications:

- - - Buprenorphine or Buprenorphine/naloxone, oral
    - Buprenorphine (extended release injectable or implant)
    - Methadone administration
    - Naltrexone (extended-release injectable)

Pharmacy claims for medications with negative, missing, or zero days’ supply were not included.

1. Count the number of hospital OUD discharges engaged with MOUD within 34 days following hospital discharge per unit of period.
2. Record time to MOUD engagement. Time to MOUD engagement is defined as the number of days between hospital discharge and the first MOUD fill.
3. As a sensitivity analysis, include oral naltrexone as MOUD.

**RATE**

Report the proportion (%) of community hospital OUD discharges engaged with MOUD within 34 days following hospital discharge.

- For each month during months 13-24 of the intervention
- Overall, during months 13-24 of the intervention
- Similar monthly and overall rates during pre-implementation (-12 randomization (-1)), implementation Year 1 (1-12), and post implementation (25-36*).

**EXPLORATORY MEASURE**

Report the distribution of time to MOUD engagement.

**STRATIFICATION**

Measure results may be stratified by:

- - - Age at the end of the measurement period: 18-34, 35-44, 45-54, 55-64 years
    - Gender: Male, Female
    - Race Ethnicity: Non-Hispanic White, Non-Hispanic Black, Hispanic, Others, Unknown/Missing
    - Eligibility Group: Disabled, Children, Expansion Adults, Non-Disabled Adults/Pregnancy
    - Living Area: Urban, Rural
    - Hospital ID: TBD
    - Intervention Arm
    - Comorbidities (see below)

Measure results among the following subgroup of patients:

- **Any Infectious diseases**
  - Hepatitis C (HCV)
  - Hepatitis B (HBV)
  - HIV
- **Any Mental illness and substance use disorders**
  - Anxiety disorder
  - Mood disorder
  - Schizophrenic and other psychotic disorders
  - PTSD
  - Other SUD (excludes opioid-related disorders, poisoning codes, and remission codes):

alcohol use disorder, cannabis use disorder, cocaine use disorder, other psychoactive substance use disorder, amphetamine-type stimulant use disorder, others (sedative/hypnotic/anxiolytic related disorders, hallucinogen-related disorders, any pregnancy related SUD, inhalant-related disorders)

- **Any Other opioid-related medical complications**
  - Intracranial and intraspinal abscess
  - Osteomyelitis
  - Endocarditis
  - Soft skin tissue infection

*Appendix - Diagnosis codes for comorbidities*

**Hepatitis C**

if Diag(i) in ('B182','B1710','B1711','B1920','B1921')

then HCV=1;

**Hepatitis B**

if Diag(i) in (‘B160’, ‘B161’, ‘B162’, ‘B169’, ‘B170’, ‘B180’, ‘B181’, ‘B1910’, ‘B1911’)

then HBV=1;

**HIV**

If Diag(i) in (‘O98711’, ‘O98712’, ‘O98713’, ‘O98719’, ‘O9872’, ‘O9873’, ‘B20’, ‘Z21’)

then HIV=1; from Marian’s project

**Anxiety disorder**

If Diag(i) in ( 'F064', 'F4000', 'F4001', 'F4002', 'F4010', 'F4011', 'F40210', 'F40218', 'F40220', 'F40228', 'F40230', 'F40231', 'F40232', 'F40233', 'F40240', 'F40241', 'F40242', 'F40243', 'F40248', 'F40290', 'F40291', 'F40298', 'F408', 'F409', 'F410', 'F411', 'F413', 'F418', 'F419', 'F42', 'F430', 'F4310', 'F4311', 'F4312', 'F449', 'F458', 'F488', 'F489', 'F938', 'F99', 'R452', 'R455', 'R456', 'R457')

then anxiety_disorder =1; from RAMP

**Mood disorder**

If Diag(i) in ('F320', 'F321', 'F322', 'F323', 'F324', 'F325', 'F329', 'F330', 'F331', 'F332', 'F333', 'F3340', 'F3341', 'F3342', 'F339', 'F341', 'F3010', 'F3011', 'F3012', 'F3013', 'F302', 'F303', 'F304', 'F308', 'F309', 'F310', 'F3110', 'F3111', 'F3112', 'F3113', 'F312', 'F3130', 'F3131', 'F3132', 'F314', 'F315', 'F3160', 'F3161', 'F3162', 'F3163', 'F3164', 'F3170', 'F3171', 'F3172', 'F3173', 'F3174', 'F3175', 'F3176', 'F3177', 'F3178', 'F3181', 'F3189', 'F319', 'F328', 'F338', 'F348', 'F349', 'F39', 'F0630')

then mood_disorder =1; from RAMP

**Schizophrenic and other psychotic disorders**

if Diag(i) in ('F060', 'F062','F200','F201', 'F202', 'F203', 'F205', 'F2081', 'F2089', 'F209', 'F22','F23', 'F24','F250', 'F251','F258','F259', 'F28','F29','F323','F333', 'F4489')

then schizo_other_psych=1; from RAMP

**PTSD**

if Diag(i) in ('F4310’, ‘F4311’, ‘F4312’)

then PTSD=1; from Khadejah Mahmoud

**Opioid-related diseases** (from Dylan Nagy, opioid dashboard project)

- intracranial and intraspinal abscess

if Diag(i) in ('G060', 'G061', 'G062', 'G07') then abscess=1;

- osteomyelitis

if Diag(i) in ('M8600', 'M8610', 'M8620', 'M86011', 'M86012', 'M86019', 'M86111', 'M86112', 'M86119', 'M86211', 'M86212', 'M86219', 'M86021', 'M86022', 'M86029', 'M86121', 'M86122', 'M86129', 'M86221', 'M86222', 'M86229', 'M86031', 'M86032', 'M86039', 'M86131', 'M86132', 'M86139', 'M86231', 'M86232', 'M86239', 'M86041', 'M86042', 'M86049', 'M86141', 'M86142', 'M86149', 'M86241', 'M86242', 'M86249', 'M86051', 'M86052', 'M86059', 'M86151', 'M86152', 'M86159', 'M86251', 'M86252', 'M86259', 'M86061', 'M86062', 'M86069', 'M86161', 'M86162', 'M86169', 'M86261', 'M86262', 'M86269', 'M86071', 'M86072', 'M86079', 'M86171', 'M86172', 'M86179', 'M86271', 'M86272', 'M86279', 'M8608', 'M8618', 'M8628', 'M8609', 'M8619', 'M8629', 'M869', 'M4620', 'M4621', 'M4622', 'M4623', 'M4624', 'M4625', 'M4626', 'M4627', 'M4628')

then osteomyelitis=1;

- endocarditis

if Diag(i) in ('I330', 'I39', 'I339') then endocarditis=1;

- soft skin tissue infections

if Diag(i) in ('L02511', 'L02512', 'L02519', 'L03113', 'L03114', 'L03119', 'L03123', 'L03124', 'L03129', 'L0231', 'L03317', 'L03327', 'L02415', 'L02416', 'L02419', 'L03115', 'L03116', 'L03125', 'L03126', 'L02611', 'L02612', 'L02619', 'L02811', 'L02818', 'L03811', 'L03818', 'L03891', 'L03898', 'L0291', 'L0390', 'L0391', 'L983', 'L040', 'L041', 'L042', 'L043', 'L048', 'L049', 'L0100', 'L0101', 'L0102', 'L0103', 'L0109', 'L011', 'L0501', 'L0502', 'L0591', 'L0592', 'L080', 'L88', 'L0881', 'L0889', 'L928', 'L980', 'B781', 'E832', 'L0882', 'L089', 'L8990', 'L8991', 'L8992', 'L8993', 'L8994', 'L8995', 'L89000', 'L89001', 'L89002', 'L89003', 'L89004', 'L89009', 'L89010', 'L89011', 'L89012', 'L89013', 'L89014', 'L89019', 'L89020', 'L89021', 'L89022', 'L89023', 'L89024', 'L89029', 'L89100', 'L89101', 'L89102', 'L89103', 'L89104', 'L89109', 'L89110', 'L89111', 'L89112', 'L89113', 'L89114', 'L89119', 'L89120', 'L89121', 'L89122', 'L89123', 'L89124', 'L89129', 'L89130', 'L89131', 'L89132', 'L89133', 'L89134', 'L89139', 'L89140', 'L89141', 'L89142', 'L89143', 'L89144', 'L89149', 'L89150', 'L89151', 'L89152', 'L89153', 'L89154', 'L89159', 'L8940', 'L8941', 'L8942', 'L8943', 'L8944', 'L8945', 'L89200', 'L89201', 'L89202', 'L89203', 'L89204', 'L89209', 'L89210', 'L89211', 'L89212', 'L89213', 'L89214', 'L89219', 'L89220', 'L89221', 'L89222', 'L89223', 'L89224', 'L89229', 'L89300', 'L89301', 'L89302', 'L89303', 'L89304', 'L89309', 'L89310', 'L89311', 'L89312', 'L89313', 'L89314', 'L89319', 'L89320', 'L89321', 'L89322', 'L89323', 'L89324', 'L89329', 'L89500', 'L89501', 'L89502', 'L89503', 'L89504', 'L89509', 'L89510', 'L89511', 'L89512', 'L89513', 'L89514', 'L89519', 'L89520', 'L89521', 'L89522', 'L89523', 'L89524', 'L89529', 'L89600', 'L89601', 'L89602', 'L89603', 'L89604', 'L89609', 'L89610', 'L89611', 'L89612', 'L89613', 'L89614', 'L89619', 'L89620', 'L89621', 'L89622', 'L89623', 'L89624', 'L89629', 'L89810', 'L89811', 'L89812', 'L89813', 'L89814', 'L89819', 'L89890', 'L89891', 'L89892', 'L89893', 'L89894', 'L89899', 'L97901', 'L97902', 'L97903', 'L97904', 'L97905', 'L97906', 'L97908', 'L97909', 'L97911', 'L97912', 'L97913', 'L97914', 'L97915', 'L97916', 'L97918', 'L97919', 'L97921', 'L97922', 'L97923', 'L97924', 'L97925', 'L97926', 'L97928', 'L97929', 'I70231', 'I70241', 'I70331', 'I70341', 'I70431', 'I70441', 'I70531', 'I70541', 'I70631', 'I70641', 'I70731', 'I70741', 'L97101', 'L97102', 'L97103', 'L97104', 'L97105', 'L97106', 'L97108', 'L97109', 'L97111', 'L97112', 'L97113', 'L97114', 'L97115', 'L97116', 'L97118', 'L97119', 'L97121', 'L97122', 'L97123', 'L97124', 'L97125', 'L97126', 'L97128', 'L97129', 'I70232', 'I70242', 'I70332', 'I70342', 'I70432', 'I70442', 'I70532', 'I70542', 'I70632', 'I70642', 'I70732', 'I70742', 'L97201', 'L97202', 'L97203', 'L97204', 'L97205', 'L97206', 'L97208', 'L97209', 'L97211', 'L97212', 'L97213', 'L97214', 'L97215', 'L97216', 'L97218', 'L97219', 'L97221', 'L97222', 'L97223', 'L97224', 'L97225', 'L97226', 'L97228', 'L97229', 'I70233', 'I70243', 'I70333', 'I70343', 'I70433', 'I70443', 'I70533', 'I70543', 'I70633', 'I70643', 'I70733', 'I70743', 'L97301', 'L97302', 'L97303', 'L97304', 'L97305', 'L97306', 'L97308', 'L97309', 'L97311', 'L97312', 'L97313', 'L97314', 'L97315', 'L97316', 'L97318', 'L97319', 'L97321', 'L97322', 'L97323', 'L97324', 'L97325', 'L97326', 'L97328', 'L97329', 'I70234', 'I70244', 'I70334', 'I70344', 'I70434', 'I70444', 'I70534', 'I70544', 'I70634', 'I70644', 'I70734', 'I70744', 'L97401', 'L97402', 'L97403', 'L97404', 'L97405', 'L97406', 'L97408', 'L97409', 'L97411', 'L97412', 'L97413', 'L97414', 'L97415', 'L97416', 'L97418', 'L97419', 'L97421', 'L97422', 'L97423', 'L97424', 'L97425', 'L97426', 'L97428', 'L97429', 'I70235', 'I70245', 'I70335', 'I70345', 'I70435', 'I70445', 'I70535', 'I70545', 'I70635', 'I70645', 'I70735', 'I70745', 'L97501', 'L97502', 'L97503', 'L97504', 'L97505', 'L97506', 'L97508', 'L97509', 'L97511', 'L97512', 'L97513', 'L97514', 'L97515', 'L97516', 'L97518', 'L97519', 'L97521', 'L97522', 'L97523', 'L97524', 'L97525', 'L97526', 'L97528', 'L97529', 'I70238', 'I70239', 'I70248', 'I70249', 'I70338', 'I70339', 'I70348', 'I70349', 'I70438', 'I70439', 'I70448', 'I70449', 'I70538', 'I70539', 'I70548', 'I70549', 'I70638', 'I70639', 'I70648', 'I70649', 'I70738', 'I70739', 'I70748', 'I70749', 'L97801', 'L97802', 'L97803', 'L97804', 'L97805', 'L97806', 'L97808', 'L97809', 'L97811', 'L97812', 'L97813', 'L97814', 'L97815', 'L97816', 'L97818', 'L97819', 'L97821', 'L97822', 'L97823', 'L97824', 'L97825', 'L97826', 'L97828', 'L97829', 'I7025', 'I7035', 'I7045', 'I7055', 'I7065', 'I7075', 'L98411', 'L98412', 'L98413', 'L98414', 'L98415', 'L98416', 'L98418', 'L98419', 'L98421', 'L98422', 'L98423', 'L98424', 'L98425', 'L98426', 'L98428', 'L98429', 'L98491', 'L98492', 'L98493', 'L98494', 'L98495', 'L98496', 'L98498', 'L98499', 'M726', 'E0852', 'E0952', 'E1052', 'E1152', 'I70361', 'I70362', 'I70363', 'I70368', 'I70369', 'I70461', 'I70462', 'I70463', 'I70468', 'I70469', 'I70561', 'I70562', 'I70563', 'I70568', 'I70569', 'I70661', 'I70662', 'I70663', 'I70668', 'I70669', 'I70761', 'I70762', 'I70763', 'I70768', 'I70769', 'I7301', 'I96', 'T798XXA', 'T827XXA', 'T8740', 'T8741', 'T8742', 'T8743', 'T8744', 'T814XXA', 'K6811', 'K610', 'K611', 'K612', 'K613', 'K614', 'L0202', 'L0203', 'L0212', 'L0213', 'L02221', 'L02222', 'L02223', 'L02224', 'L02225', 'L02226', 'L02229', 'L02231', 'L02232', 'L02233', 'L02234', 'L02235', 'L02236', 'L02239', 'L02421', 'L02422', 'L02423', 'L02424', 'L02429', 'L02431', 'L02432', 'L02433', 'L02434', 'L02439', 'L02521', 'L02522', 'L02529', 'L02531', 'L02532', 'L02539', 'L0232', 'L0233', 'L02425', 'L02426', 'L02435', 'L02436', 'L02621', 'L02622', 'L02629', 'L02631', 'L02632', 'L02639', 'L02821', 'L02828', 'L02831', 'L02838', 'L0292', 'L0293', 'L03011', 'L03012', 'L03019', 'L03021', 'L03022', 'L03029', 'L03031', 'L03032', 'L03039', 'L03041', 'L03042', 'L03049', 'K122', 'L0201', 'L03211', 'L03212', 'L03213', 'L0211', 'L03221', 'L03222', 'L02211', 'L02212', 'L02213', 'L02214', 'L02215', 'L02216', 'L02219', 'L03311', 'L03312', 'L03313', 'L03314', 'L03315', 'L03316', 'L03319', 'L03321', 'L03322', 'L03323', 'L03324', 'L03325', 'L03326', 'L03329', 'L02411', 'L02412', 'L02413', 'L02414', 'L03111', 'L03112', 'L03121', 'L03122') then ssti=1;

**Other SUD**

‘MACPAC SUD_codes_no_nicotine_tobacco_no_poisoning_July2021.xlsx’ (exclude if remission=1)

- - Alcohol use disorder (‘F10’)
  - Cannabis use disorder (‘F12’)
  - Cocaine use disorder (‘F14’)
  - Amphetamine-type stimulant use disorder (‘F15’)
  - Other psychoactive substance related disorders (‘F19’)
  - Other:
    - Any Sedative, hypnotic, or anxiolytic related disorders (‘F13’)
    - Any pregnancy related SUD (‘O’ codes in the list)
    - Hallucinogen related disorders (‘F16’)
    - Inhalant related disorders (‘F18’)
    - Any other SUD (misc)

**Analysis Plan: CTN Secondary and Exploratory Measures**

**November 18, 2022**

**Measure Description (1-3 secondary; 4-6 exploratory measures):**

1. The proportion of discharges with any subsequent ED visit or acute care hospitalization (not transfer) within 30 days of index hospital discharge during months 13-24 of intervention.
2. The proportion of discharges with any subsequent ED visit or acute care hospitalization (not transfer) within 30 days of index hospital discharge that are opioid-related during months 13-24 of intervention.
3. The proportion of discharges against medical advice during the index hospitalization months 13-24 of intervention
4. Deaths (all-cause) rates in the 30-day period after hospital OUD discharge, and time to death
5. Index hospitalization length of stay
6. Non-fatal opioid-related overdose

The measures are also calculated for each month of pre-implementation (-12 randomization (-1)), implementation Year 1 (1-12), implementation Year 2 (13-24), and post implementation (25-36*).

**DENOMINATOR:** Hospital discharges with OUD diagnosis from all non-dual, full-benefit Medicaid enrollees who are 18-64 years of age

CREATE DENOMINATOR:

*Note: This is the same as denominator for primary measure, MOUD Engagement within 34 Days Post-Discharge.*

1. For months 13-24 of the intervention, identify non-dual, full-benefit Medicaid enrollees who are 18-64 years of age for the duration of the year

*Note: This is consistent with the Common data Model (CDM), which only includes non-dual, full-benefit Medicaid enrollees who are 18 years of age and older for the duration of the year.*

1. For individuals identified in Step 1, identify all inpatient encounters with any diagnosis (all diagnosis fields) of OUD at any time during the measurement period.

The ICD-10 diagnosis codes used to identify OUD included:

1. Of inpatient encounters identified in Step 2, exclude those billed from a non-participating hospital.
2. Identify unique hospital stays. We’ll treat inpatient claims as a single hospital stay if they are from same hospital AND the DOS_END from the first stay precedes the DOS_START to a subsequent stay by one calendar day or less.
3. Exclude hospital stays where the enrollee died during the stay.
4. Exclude hospital stays if the enrollee had a MOUD in the 14 days prior to hospital stay.
5. Check continuous enrollment from date of discharge to 33 days after hospital discharge (total of 34 days). Exclude those without the continuous enrollment.
6. Flag if there is a direct transfer to non-participating hospital after hospital stay at a participating hospital. A direct transfer is when the discharge date from the initial stay precedes the admission date to a subsequent stay by one calendar day or less.
7. Count the number of hospital discharges with OUD diagnosis per unit of period. This is the denominator.

**NUMERATOR:**

- Hospital OUD discharges with any subsequent ED visit or acute care hospitalization (not transfer) within 30 days of index hospital discharge
- Hospital OUD discharges with any subsequent ED visit or acute care hospitalization (not transfer) within 30 days of index hospital discharge that are opioid-related
- Hospital OUD discharges against medical advice
- Deaths (all-cause) in the 30-day period after hospital OUD discharge (without Step 7 in denominator)
- Non-fatal opioid-related overdose within 30/60/90 days of index hospital discharge

CREATE NUMERATOR:

1. Of observations in the denominator, identify those with at least one ED visit (ED Value Set*) within 30 days beginning on the day of hospital discharge. Exclude ED visits that lead to acute care hospitalization (i.e., Exclude if there is any acute hospitalization within one day from ED visit).

ED Value Set*:

CPT: “99281”, “99282”, “99283”, “99284”, “99285"

UBREV: “0450”, “0451”, “0452”, “0456”, “0459”, “0981”

1. Of observations in the denominator, identify those with at least one acute care hospitalization within 30 days beginning on the day of hospital discharge. Exclude direct transfer.
   1. Identify all acute and non-acute inpatient discharges (Inpatient Stay Value Set*)

UBREV: "0100”, “0101”, “0110”, “0111”, “0112”, “0113”, “0114”, “0116”, “0117”, “0118”, “0119”, “0120”, “0121”, “0122”, "0123”, “0124”, “0126”, “0127”, “0128”, “0129”, “0130”, “0131”, “0132”, “0133”, “0134”, “0136”, “0137”, “0138”, "0139”, “0140”, “0141”, “0142”, “0143”, “0144”, “0146”, “0147”, “0148”, “0149”, “0150”, “0151”, “0152”, “0153”, "0154”, “0156”, “0157”, “0158”, “0159”, “0160”, “0164”, “0167”, “0169”, “0170”, “0171”, “0172”, “0173”, “0174”, "0179”, “0190”, “0191”, “0192”, “0193”, “0194”, “0199”, “0200”, “0201”, “0202”, “0203”, “0204”, “0206”, “0207”, "0208”, “0209”, “0210”, “0211”, “0212”, “0213”, “0214”, “0219”, “1000”, “1001”, “1002"

- 1. Exclude non-acute inpatient stays (Nonacute Inpatient Stay Value Set*)

UBREV: “0022”, “0024”, “0118”, “0128”, “0138”, “0148”, “0158”, “0190”, “0191”, “0192”, “0193”, “0194”, “0199”, “0524”, “0525”, “0550”, “0551”, “0552”, “0559”, “0660”, “0661”, “0662”, “0663”, “0669”, “1000”, “1001”, “1002”

Note: There is another HEDIS value set Acute Inpatient Value Set* used to identify acute inpatient encounters.

CPT: “99221”, “99222”, “99223”, “99231”, “99232”, “99233”, “99238”, “99239”, “99251”, “99252”, “99253”, “99254”, “99255”, “99291”

- 1. Exclude direct transfers.

1. Of observations in the denominator, identify those with at least one ED visit (ED Value Set*) with a diagnosis of OUD within 30 days beginning on the day of hospital discharge that are opioid-related. The OUD diagnosis must be on the ED claim. Exclude ED visits that lead to acute care hospitalization.
2. Of observations in the denominator, identify those with at least one acute care hospitalization with a diagnosis of OUD within 30 days beginning on the day of hospital discharge that are opioid-related. The OUD diagnosis must be on the claim for acute care inpatient claim. Exclude direct transfer.
3. Of observations in the denominator, identify discharges against medical advice. The discharge against medical advice is identified with DISCHARGE_STATUS=’07’ on hospitalization discharge claim.
4. Of observations in the denominator, identify enrollees who died in the 30-day period after hospital OUD discharge (i.e., DOS_END (discharge date) < DOD <= DOS_END+30).
5. Of observations in the denominator, identify those with non-fatal opioid-related overdose within x days of index hospital discharge. Opioid-related overdose can be identified via ICD-10 diagnosis codes listed in 'opioid_overdose_poisoning_Green.xlsx.' Exclude observations with DOD within 30 days of overdose claim.
6. For each #1-#7, count the number of qualifying discharges. These are the numerators.

**All the Value Sets are in ‘2021-Adult-HEDIS-VSD_codes_only.xlsx’*

**RATE**

Report the following proportions:

1. Proportion of discharges with any subsequent ED visit (all-cause) within 30 days of index hospital discharge.
2. Proportion of discharges with any acute care hospitalization (all-cause) within 30 days of index hospital discharge.
3. Proportion of discharges with any subsequent opioid-related ED visit within 30 days of index hospital discharge.
4. Proportion of discharges with any opioid-related acute care hospitalization within 30 days of index hospital discharge.
5. Proportion of discharges against medical advice during the index hospitalization
6. Proportion of discharges with all-cause death within 30 days of index hospital discharge.
7. Proportion of discharges with non-fatal opioid-related overdose within x days of index hospital discharge.

All proportions are calculated:

- For each month during months 13-24 of the intervention
- Overall, during months 13-24 of the intervention
- Similar monthly and overall rates during pre-implementation (-12 randomization (-1)), implementation Year 1 (1-12), and post implementation (25-36*).

**OTHER SECONDARY MEASURES** (report distributions in days, for each month and overall)**:**

1. Time to death among those died (all-cause) in the 30-day period after hospital OUD discharge (i.e., DOD-DOS_END)
2. Index hospitalization length of stay (i.e., DOS_END-DOS_START from unique hospital stays identified as the denominator)

**STRATIFICATION**

Measure results may be stratified by:

- - 1. Age at the end of the measurement period: 18-34, 35-44, 45-54, 55-64 years
    2. Gender: Male, Female
    3. Race Ethnicity: Non-Hispanic White, Non-Hispanic Black, Hispanic, Others, Unknown/Missing
    4. Eligibility Group: Disabled, Children, Expansion Adults, Non-Disabled Adults/Pregnancy
    5. Living Area: Urban, Rural
    6. Hospital ID: TBD
    7. Intervention Arm
    8. Comorbidities (see below)

Secondary measure results among the following subgroup of patients:

- **Any Infectious diseases**
  - Hepatitis C (HCV)
  - Hepatitis B (HBV)
  - HIV
- **Any Mental illness and substance use disorders**
  - Anxiety disorder
  - Mood disorder
  - Schizophrenic and other psychotic disorders
  - PTSD
  - Other SUD (excludes opioid-related disorders, poisoning codes, and remission codes):

alcohol use disorder, cannabis use disorder, cocaine use disorder, other psychoactive substance use disorder, amphetamine-type stimulant use disorder, others (sedative/hypnotic/anxiolytic related disorders, hallucinogen-related disorders, any pregnancy related SUD, inhalant-related disorders)

- **Any Other opioid-related medical complications**
  - Intracranial and intraspinal abscess
  - Osteomyelitis
  - Endocarditis
  - Soft skin tissue infection

*Appendix - Diagnosis codes for comorbidities*

if Diag(i) in ('B182','B1710','B1711','B1920','B1921','Z2252')

then HCV=1;

if Diag(i) in (‘B160’, ‘B161’, ‘B162’, ‘B169’, ‘B170’, ‘B180’, ‘B181’, ‘B1910’, ‘B1911’)

then HBV=1;

**Anxiety disorder**

If Diag(i) in ( 'F064', 'F4000', 'F4001', 'F4002', 'F4010', 'F4011', 'F40210', 'F40218', 'F40220', 'F40228', 'F40230', 'F40231', 'F40232', 'F40233', 'F40240', 'F40241', 'F40242', 'F40243', 'F40248', 'F40290', 'F40291', 'F40298', 'F408', 'F409', 'F410', 'F411', 'F413', 'F418', 'F419', 'F42', 'F430', 'F4310', 'F4311', 'F4312', 'F449', 'F458', 'F488', 'F489', 'F938', 'F99', 'R452', 'R455', 'R456', 'R457')

then anxiety_disorder =1; from RAMP

**Mood disorder**

If Diag(i) in ('F320', 'F321', 'F322', 'F323', 'F324', 'F325', 'F329', 'F330', 'F331', 'F332', 'F333', 'F3340', 'F3341', 'F3342', 'F339', 'F341', 'F3010', 'F3011', 'F3012', 'F3013', 'F302', 'F303', 'F304', 'F308', 'F309', 'F310', 'F3110', 'F3111', 'F3112', 'F3113', 'F312', 'F3130', 'F3131', 'F3132', 'F314', 'F315', 'F3160', 'F3161', 'F3162', 'F3163', 'F3164', 'F3170', 'F3171', 'F3172', 'F3173', 'F3174', 'F3175', 'F3176', 'F3177', 'F3178', 'F3181', 'F3189', 'F319', 'F328', 'F338', 'F348', 'F349', 'F39', 'F0630')

then mood_disorder =1; from RAMP

**Schizophrenic and other psychotic disorders**

if Diag(i) in ('F060', 'F062','F200','F201', 'F202', 'F203', 'F205', 'F2081', 'F2089', 'F209', 'F22','F23', 'F24','F250', 'F251','F258','F259', 'F28','F29','F323','F333', 'F4489')

then schizo_other_psych=1; from RAMP

**PTSD**

if Diag(i) in ('F4310’, ‘F4311’, ‘F4312’)

then PTSD=1; from Khadejah Mahmoud

**Opioid-related diseases** (from Dylan Nagy, opioid dashboard project)

- intracranial and intraspinal abscess

if Diag(i) in ('G060', 'G061', 'G062', 'G07') then abscess=1;

- osteomyelitis

if Diag(i) in ('M8600', 'M8610', 'M8620', 'M86011', 'M86012', 'M86019', 'M86111', 'M86112', 'M86119', 'M86211', 'M86212', 'M86219', 'M86021', 'M86022', 'M86029', 'M86121', 'M86122', 'M86129', 'M86221', 'M86222', 'M86229', 'M86031', 'M86032', 'M86039', 'M86131', 'M86132', 'M86139', 'M86231', 'M86232', 'M86239', 'M86041', 'M86042', 'M86049', 'M86141', 'M86142', 'M86149', 'M86241', 'M86242', 'M86249', 'M86051', 'M86052', 'M86059', 'M86151', 'M86152', 'M86159', 'M86251', 'M86252', 'M86259', 'M86061', 'M86062', 'M86069', 'M86161', 'M86162', 'M86169', 'M86261', 'M86262', 'M86269', 'M86071', 'M86072', 'M86079', 'M86171', 'M86172', 'M86179', 'M86271', 'M86272', 'M86279', 'M8608', 'M8618', 'M8628', 'M8609', 'M8619', 'M8629', 'M869', 'M4620', 'M4621', 'M4622', 'M4623', 'M4624', 'M4625', 'M4626', 'M4627', 'M4628')

then osteomyelitis=1;

- endocarditis

if Diag(i) in ('I330', 'I39', 'I339') then endocarditis=1;

- soft skin tissue infections

if Diag(i) in ('L02511', 'L02512', 'L02519', 'L03113', 'L03114', 'L03119', 'L03123', 'L03124', 'L03129', 'L0231', 'L03317', 'L03327', 'L02415', 'L02416', 'L02419', 'L03115', 'L03116', 'L03125', 'L03126', 'L02611', 'L02612', 'L02619', 'L02811', 'L02818', 'L03811', 'L03818', 'L03891', 'L03898', 'L0291', 'L0390', 'L0391', 'L983', 'L040', 'L041', 'L042', 'L043', 'L048', 'L049', 'L0100', 'L0101', 'L0102', 'L0103', 'L0109', 'L011', 'L0501', 'L0502', 'L0591', 'L0592', 'L080', 'L88', 'L0881', 'L0889', 'L928', 'L980', 'B781', 'E832', 'L0882', 'L089', 'L8990', 'L8991', 'L8992', 'L8993', 'L8994', 'L8995', 'L89000', 'L89001', 'L89002', 'L89003', 'L89004', 'L89009', 'L89010', 'L89011', 'L89012', 'L89013', 'L89014', 'L89019', 'L89020', 'L89021', 'L89022', 'L89023', 'L89024', 'L89029', 'L89100', 'L89101', 'L89102', 'L89103', 'L89104', 'L89109', 'L89110', 'L89111', 'L89112', 'L89113', 'L89114', 'L89119', 'L89120', 'L89121', 'L89122', 'L89123', 'L89124', 'L89129', 'L89130', 'L89131', 'L89132', 'L89133', 'L89134', 'L89139', 'L89140', 'L89141', 'L89142', 'L89143', 'L89144', 'L89149', 'L89150', 'L89151', 'L89152', 'L89153', 'L89154', 'L89159', 'L8940', 'L8941', 'L8942', 'L8943', 'L8944', 'L8945', 'L89200', 'L89201', 'L89202', 'L89203', 'L89204', 'L89209', 'L89210', 'L89211', 'L89212', 'L89213', 'L89214', 'L89219', 'L89220', 'L89221', 'L89222', 'L89223', 'L89224', 'L89229', 'L89300', 'L89301', 'L89302', 'L89303', 'L89304', 'L89309', 'L89310', 'L89311', 'L89312', 'L89313', 'L89314', 'L89319', 'L89320', 'L89321', 'L89322', 'L89323', 'L89324', 'L89329', 'L89500', 'L89501', 'L89502', 'L89503', 'L89504', 'L89509', 'L89510', 'L89511', 'L89512', 'L89513', 'L89514', 'L89519', 'L89520', 'L89521', 'L89522', 'L89523', 'L89524', 'L89529', 'L89600', 'L89601', 'L89602', 'L89603', 'L89604', 'L89609', 'L89610', 'L89611', 'L89612', 'L89613', 'L89614', 'L89619', 'L89620', 'L89621', 'L89622', 'L89623', 'L89624', 'L89629', 'L89810', 'L89811', 'L89812', 'L89813', 'L89814', 'L89819', 'L89890', 'L89891', 'L89892', 'L89893', 'L89894', 'L89899', 'L97901', 'L97902', 'L97903', 'L97904', 'L97905', 'L97906', 'L97908', 'L97909', 'L97911', 'L97912', 'L97913', 'L97914', 'L97915', 'L97916', 'L97918', 'L97919', 'L97921', 'L97922', 'L97923', 'L97924', 'L97925', 'L97926', 'L97928', 'L97929', 'I70231', 'I70241', 'I70331', 'I70341', 'I70431', 'I70441', 'I70531', 'I70541', 'I70631', 'I70641', 'I70731', 'I70741', 'L97101', 'L97102', 'L97103', 'L97104', 'L97105', 'L97106', 'L97108', 'L97109', 'L97111', 'L97112', 'L97113', 'L97114', 'L97115', 'L97116', 'L97118', 'L97119', 'L97121', 'L97122', 'L97123', 'L97124', 'L97125', 'L97126', 'L97128', 'L97129', 'I70232', 'I70242', 'I70332', 'I70342', 'I70432', 'I70442', 'I70532', 'I70542', 'I70632', 'I70642', 'I70732', 'I70742', 'L97201', 'L97202', 'L97203', 'L97204', 'L97205', 'L97206', 'L97208', 'L97209', 'L97211', 'L97212', 'L97213', 'L97214', 'L97215', 'L97216', 'L97218', 'L97219', 'L97221', 'L97222', 'L97223', 'L97224', 'L97225', 'L97226', 'L97228', 'L97229', 'I70233', 'I70243', 'I70333', 'I70343', 'I70433', 'I70443', 'I70533', 'I70543', 'I70633', 'I70643', 'I70733', 'I70743', 'L97301', 'L97302', 'L97303', 'L97304', 'L97305', 'L97306', 'L97308', 'L97309', 'L97311', 'L97312', 'L97313', 'L97314', 'L97315', 'L97316', 'L97318', 'L97319', 'L97321', 'L97322', 'L97323', 'L97324', 'L97325', 'L97326', 'L97328', 'L97329', 'I70234', 'I70244', 'I70334', 'I70344', 'I70434', 'I70444', 'I70534', 'I70544', 'I70634', 'I70644', 'I70734', 'I70744', 'L97401', 'L97402', 'L97403', 'L97404', 'L97405', 'L97406', 'L97408', 'L97409', 'L97411', 'L97412', 'L97413', 'L97414', 'L97415', 'L97416', 'L97418', 'L97419', 'L97421', 'L97422', 'L97423', 'L97424', 'L97425', 'L97426', 'L97428', 'L97429', 'I70235', 'I70245', 'I70335', 'I70345', 'I70435', 'I70445', 'I70535', 'I70545', 'I70635', 'I70645', 'I70735', 'I70745', 'L97501', 'L97502', 'L97503', 'L97504', 'L97505', 'L97506', 'L97508', 'L97509', 'L97511', 'L97512', 'L97513', 'L97514', 'L97515', 'L97516', 'L97518', 'L97519', 'L97521', 'L97522', 'L97523', 'L97524', 'L97525', 'L97526', 'L97528', 'L97529', 'I70238', 'I70239', 'I70248', 'I70249', 'I70338', 'I70339', 'I70348', 'I70349', 'I70438', 'I70439', 'I70448', 'I70449', 'I70538', 'I70539', 'I70548', 'I70549', 'I70638', 'I70639', 'I70648', 'I70649', 'I70738', 'I70739', 'I70748', 'I70749', 'L97801', 'L97802', 'L97803', 'L97804', 'L97805', 'L97806', 'L97808', 'L97809', 'L97811', 'L97812', 'L97813', 'L97814', 'L97815', 'L97816', 'L97818', 'L97819', 'L97821', 'L97822', 'L97823', 'L97824', 'L97825', 'L97826', 'L97828', 'L97829', 'I7025', 'I7035', 'I7045', 'I7055', 'I7065', 'I7075', 'L98411', 'L98412', 'L98413', 'L98414', 'L98415', 'L98416', 'L98418', 'L98419', 'L98421', 'L98422', 'L98423', 'L98424', 'L98425', 'L98426', 'L98428', 'L98429', 'L98491', 'L98492', 'L98493', 'L98494', 'L98495', 'L98496', 'L98498', 'L98499', 'M726', 'E0852', 'E0952', 'E1052', 'E1152', 'I70361', 'I70362', 'I70363', 'I70368', 'I70369', 'I70461', 'I70462', 'I70463', 'I70468', 'I70469', 'I70561', 'I70562', 'I70563', 'I70568', 'I70569', 'I70661', 'I70662', 'I70663', 'I70668', 'I70669', 'I70761', 'I70762', 'I70763', 'I70768', 'I70769', 'I7301', 'I96', 'T798XXA', 'T827XXA', 'T8740', 'T8741', 'T8742', 'T8743', 'T8744', 'T814XXA', 'K6811', 'K610', 'K611', 'K612', 'K613', 'K614', 'L0202', 'L0203', 'L0212', 'L0213', 'L02221', 'L02222', 'L02223', 'L02224', 'L02225', 'L02226', 'L02229', 'L02231', 'L02232', 'L02233', 'L02234', 'L02235', 'L02236', 'L02239', 'L02421', 'L02422', 'L02423', 'L02424', 'L02429', 'L02431', 'L02432', 'L02433', 'L02434', 'L02439', 'L02521', 'L02522', 'L02529', 'L02531', 'L02532', 'L02539', 'L0232', 'L0233', 'L02425', 'L02426', 'L02435', 'L02436', 'L02621', 'L02622', 'L02629', 'L02631', 'L02632', 'L02639', 'L02821', 'L02828', 'L02831', 'L02838', 'L0292', 'L0293', 'L03011', 'L03012', 'L03019', 'L03021', 'L03022', 'L03029', 'L03031', 'L03032', 'L03039', 'L03041', 'L03042', 'L03049', 'K122', 'L0201', 'L03211', 'L03212', 'L03213', 'L0211', 'L03221', 'L03222', 'L02211', 'L02212', 'L02213', 'L02214', 'L02215', 'L02216', 'L02219', 'L03311', 'L03312', 'L03313', 'L03314', 'L03315', 'L03316', 'L03319', 'L03321', 'L03322', 'L03323', 'L03324', 'L03325', 'L03326', 'L03329', 'L02411', 'L02412', 'L02413', 'L02414', 'L03111', 'L03112', 'L03121', 'L03122') then ssti=1;

**Other SUD**

‘MACPAC SUD_codes_no_nicotine_tobacco_no_poisoning_July2021.xlsx’ (exclude if remission=1)

- 1. Alcohol use disorder (‘F10’)
  2. Cannabis use disorder (‘F12’)
  3. Cocaine use disorder (‘F14’)
  4. Amphetamine-type stimulant use disorder (‘F15’)
  5. Other psychoactive substance related disorders (‘F19’)
  6. Other:
     1. Any Sedative, hypnotic, or anxiolytic related disorders (‘F13’)
     2. Any pregnancy related SUD (‘O’ codes in the list)
     3. Hallucinogen related disorders (‘F16’)
     4. Inhalant related disorders (‘F18’)
     5. Any other SUD (misc)

Demographic survey

*[Logo]*

Are you associated with ${e://Field/HOSPITAL}? 🞏 No 🞏 Yes

*[Logo]*

We are asking hospital staff to complete a short survey regarding the implementation of an HBOT program at your hospital.

Your survey responses are confidential. This means that your responses will not be seen by anyone outside of the research team. Only anonymous data (that does not identify you) will be reported.

If you are not able to complete this survey in one sitting, you may save it and return to the survey **within one week using the same device**.

If you have questions or comments, contact the research team.

1. Provide the following information to construct a unique identifier. You will be asked to provide this same information if you complete additional surveys for this study in the future *(see below for example):*

Provide the following information to construct a unique identifier. If you have previously completed surveys for this study, provide the same information *(see below for example):*

Unique ID example: John Smith was born in New Orleans on March 2^nd^. His mother’s maiden name is Miller.

First 2 letters of last name: SM

First 2 letters of birth city: NE

2 digits of birth day: 02

First 2 letters of mother’s maiden name: MI

Unique ID: SMNE02MI

- 1. First 2 letters of your last name: *(e.g., SM)* _____ *(xx)*
  2. First 2 letters of city you were born in: *(e.g., NE)* _____ *(xx)*
  3. 2 digits of your day of birth: *(e.g., 02)* _____ *(xx)*
  4. First 2 letters of mother’s maiden name: *(e.g., MI)* _____ *(xx)*

*If you do not know your mother’s maiden name, choose a different pair of letters that will be memorable to you.*

Review your responses above carefully as these cannot be viewed again or updated once you click the “next” arrow.

**DEMOGRAPHICS: Community Hospital Employees (DCH)**

1. What is your age?

18-24

25-34

35-44

45-54

55-64

65-74

75+

Prefer not to answer

1. What is your gender?

Male

Female

Transgender

Nonbinary

Other gender category

Prefer not to answer

1. If “Other gender category”, specify: _______________________________
2. Do you consider yourself to be Hispanic/Latinx?

- No
- Yes
- Prefer not to answer

1. What race do you consider yourself to be? *(Select all that apply)*

- White
- Black/African American
- Asian
- American Indian or Alaska Native
- Native Hawaiian or Pacific Islander
- Other race
- Prefer not to answer

1. If “Other race”, specify: ______
2. What is your discipline?

MD/DO

Nurse practitioner

Physician assistant

Pharmacist

Social worker

Nurse (e.g., RN, LPN)

Peer recovery coach/specialist

Other discipline

Prefer not to answer

1. If “Other discipline”, specify: ____
2. What is your clinical specialty? 🞏Prefer not to answer

|  | Primary Specialty  *(select one)* | Secondary Specialties *(select all that apply)* |
| --- | --- | --- |
| Internal Medicine: | o | o |
| Family Medicine: | o | o |
| Med/Pediatrics: | o | o |
| Addiction Medicine: | o | o |
| Pain/Palliative Care: | o | o |
| Behavioral Specialist/Psychiatry: | o | o |
| Other (specify): ______ | o | o |

1. What is your primary role?

Administrator

Hospitalist

Non-hospitalist clinician

Other clinical role

Other non-clinical role

Prefer not to answer

- 1. If “Other clinical role”, specify: ________________
  2. If “Other non-clinical role”, specify: ________________

1. Do you have a leadership or administrative role in the hospital?
   - No
   - Yes
   - Prefer not to answer
   1. At what level is your leadership/administrative role? *(Select all that apply)*
      - - Hospital
        - Department
        - Section/division
        - Unit
        - Other level
        - Prefer not to answer
      1. If “Other level”, specify: __________________
2. How many years have you been in clinical practice?

<1 year

1-5 years

6-10 years

11-15 years

16-20 years

>20 years

Not applicable

Prefer not to answer

1. How many years have you worked at this hospital?

<1 year

1-5 years

6-10 years

11-15 years

16-20 years

>20 years

Prefer not to answer

1. In a typical week, how many hours do you work at this hospital?

<20 hours

20-30 hours

31-40 hours

41-50 hours

51-60 hours

>60 hours

Prefer not to answer

1. Have you participated in quality improvement projects at this hospital? ☐ No ☐ Yes
2. Have you led quality improvement projects at this hospital? ☐ No ☐ Yes
